# Intravenous catheter-related adverse events exceed drug-related adverse events in outpatient parenteral antimicrobial therapy

**DOI:** 10.1093/jac/dky474

**Published:** 2018-11-20

**Authors:** Jonathan Underwood, Michael Marks, Steve Collins, Sarah Logan, Gabriele Pollara

**Affiliations:** 1 The Hospital for Tropical Diseases, Division of Infection, University College London Hospitals NHS Foundation Trust, London, UK; 2 Clinical Research Department, London School of Hygiene and Tropical Medicine, London, UK; 3 Division of Infection & Immunity, University College London, London, UK

## Abstract

**Background:**

Drug-related adverse events (AEs) are reported to be common amongst patients receiving outpatient parenteral antimicrobial therapy (OPAT). However, comparative data regarding intravenous (iv) catheter-related AEs are lacking.

**Objectives:**

To compare drug- and iv catheter-related AEs from a large UK OPAT centre.

**Patients and methods:**

We reviewed 544 OPAT episodes [median (IQR) age: 57 (39–71) years, 60% male, 13% with diabetes] with a median (IQR) duration of 7 (2–18) days. Clinically significant drug- and iv catheter-related AEs were calculated as a percentage of OPAT episodes with an AE and also as AEs per 1000 iv drug/catheter days.

**Results:**

Drug-related AEs complicated 13 (2.4%) OPAT episodes at 1.7 (95% CI 0.9–2.9) per 1000 drug days. Catheter-related AEs occurred more frequently, complicating 32 (5.9%) episodes at 5.7 (95% CI 4.2–7.9) per 1000 iv catheter days (*χ*^2^ test for difference in AE rate: *P *<* *0.001). Non-radiologically guided midline catheters were associated with the most frequent AEs (*n *=* *23) at 15.6 (95% CI 10.3–23.4) per 1000 iv catheter days compared with other types of iv catheters (HR 8.4, 95% CI 2.4–51.9, *P *<* *0.004), and self-administration was associated with a higher rate of catheter-related AEs at 12.0 (95% CI 6.0–23.9) per 1000 iv catheter days (HR 4.15, 95% CI 1.7–9.1, *P *=* *0.007).

**Conclusions:**

Clinically significant iv catheter-related AEs occurred more frequently than drug-related AEs, especially when using non-radiologically guided midline catheters. Regular review of the need for iv therapy and switching to oral antimicrobials when appropriate is likely to minimize OPAT-related AEs.

## Introduction

Outpatient parenteral antimicrobial therapy (OPAT) prevents admission to hospital and facilitates early discharge.^1^ The clinical efficacy of OPAT compared with inpatient care appears equivalent,^1^ placing an onus on ensuring that OPAT services are delivered safely, by quantifying the rate of adverse events (AEs), including those related to the use of intravenous (iv) catheters, and the antimicrobials administered.^2^ Both have been widely reported but often as an absolute proportion over the entirety of the OPAT care episode,^3–6^ which is overly simplistic given the heterogeneity of OPAT indications, as risk is clearly related to the duration of the OPAT care episode.^7^^,^^8^

In a recent US-based study, clinically significant drug-related AEs complicated 18% of OPAT care episodes at a rate of 2.24 events per 1000 OPAT care days. Data on iv catheter-related AEs in this cohort were not provided.^9^ A separate US cohort reported catheter-related AEs at a rate of 4.29 per 1000 OPAT days, but did not report drug-related AEs.^8^ In contrast, a large UK-based retrospective study estimated drug-related and catheter-related AEs at 3.8 and 0.2 per 1000 OPAT care days, respectively.^10^ In a smaller Swiss cohort the rate of drug-related AEs was similar (4 per 1000 OPAT treatment days) with catheter-related AEs again occurring less frequently at 2.4 per 1000 OPAT treatment days.^11^

Drug-related AEs are influenced by many factors, including the choice of antimicrobials in local formularies,^12^ and some drugs, such as vancomycin, are recognized to have relatively high rates of AEs.^13^ A risk of AEs is also associated with iv catheters: tunnelled and peripherally inserted central catheters (PICCs) extend to the superior vena cava, require radiologically guided insertion and may precipitate central venous thrombosis. Midlines extend no further than the axillary vein and some can be inserted without radiological guidance.^14^ Although midlines are reported to carry greater AE risks,^15^^,^^16^ in the OPAT setting this has not been consistently observed.^7^^,^^13^ The Swiss study only included patients using either peripheral cannulae or PICCs,^11^ and one of the UK studies only included a small proportion (<3%) of patients using midlines.^10^

In this study, we used prospective data from a large UK OPAT care centre to fully characterize the rate of both drug- and catheter-related AEs. We hypothesized that it is catheter- and not drug-related AEs that drive the greatest risk to the patient during the OPAT episode.

## Patients and methods

### Ethics

The study was approved by the Audit and Research Committee at the Hospital for Tropical Diseases, University College London Hospitals (UCLH), which stated that, as this was a retrospective review of routine clinical data being analysed for service development purposes, further formal ethics approval was not required.

### Patient cohort and data extraction

We reviewed the case records of patients who were assessed by the OPAT team at UCLH between January 2015 and September 2017. Patients were accepted onto the OPAT service only after clinical review by at least one infection specialist on any day of the week. Patients’ indication for continued iv therapy was reviewed in clinics throughout the week at clinicians’ discretion, but all OPAT patients were also reviewed weekly by a multidisciplinary team, including at least two infection specialists, where the indication for iv therapy was again reviewed and outcomes determined. Data were anonymously extracted from the OPAT electronic Clinical Infectious Diseases (elCID) database at UCLH.^17^

### Clinical definitions

OPAT outcomes were defined using the standardized National Outcome Registry System (NORS) definitions (http://opatregistry.com/). Clinically significant drug-related AEs were defined as hospital readmissions or change of OPAT antimicrobial drug owing to toxicity or *Clostridioides* (*Clostridium) difficile* infection. Catheter-related AEs were defined as hospital readmission related to an iv catheter complication or iv catheter blockage, displacement, extravasation or phlebitis requiring iv catheter change. Readmission was defined as an admission to hospital during the OPAT period.

### Statistical analysis

We calculated the duration of OPAT care episodes from the date of discharge from inpatient stay to the OPAT service. Missing catheter insertion dates were imputed to be the start of the OPAT episode, or the day following removal of the previous catheter in the case of catheter replacement. Missing catheter removal dates were imputed to be the end of the OPAT episode, or the day preceding new catheter insertion. We calculated the proportion of OPAT episodes in which a drug- or iv catheter-related AE occurred and the rate of AEs per 1000 iv catheter/drug days. We fitted a Poisson regression model to assess the risk of AEs controlling for both type of iv catheter and whether OPAT was administered by nursing staff or self-administered. Statistical analysis was performed using R v3.4.2.

## Results

### OPAT patient cohort and clinical outcomes

Over a 32 month period, the OPAT service received 781 patient referrals, of which 243 were not accepted, most commonly (158, 65%) owing to infection specialist assessment that iv antimicrobials were not indicated, including the availability of oral alternatives (Table S1, available as Supplementary data at *JAC* Online). Consequently, we present data from 538 patients resulting in 544 episodes of OPAT patient care. The median age was 57 (IQR 39–71) years, 60% of the cohort was male and 13% had diabetes mellitus. The median duration of an OPAT care episode was 7 days (IQR 2–18), but was dependent on the OPAT indications, the commonest being skin and soft tissue infections (32% of episodes) (Table S2). The three most frequent antimicrobials used were ceftriaxone, teicoplanin and ertapenem (Table S3) and midline catheters accounted for over half the total indwelling iv catheter duration (Table S4). Overall, OPAT was judged to be a full/partial success in 93% of episodes. A total of 35 patients (6.4%) required readmission and there were two deaths (0.4%).

**Table 1.** Antimicrobial- and iv catheter-related OPAT AEs

	Usage (no. of episodes)	AEs, *N* (%)^a^	AE rate per 1000 days (95% CI)^b^
Antimicrobial drugs used		antimicrobial drug-related	antimicrobial drug-related
all antimicrobials	688	13 (1.9)	1.7 (0.9–2.9)
β-lactam antimicrobials	517	10 (1.9)	1.7 (0.2–12.3)
non-β-lactam antimicrobials	171	3 (1.8)	1.2 (0.4–3.6)
iv catheters used		iv catheter-related	iv catheter-related
all catheters	576	32 (5.6)	5.7 (4.2–7.9)
non-radiologically guided midline catheters	118	23 (19.5)	15.6 (10.3–23.4)
radiologically guided midline catheters	155	21 (13.5)	0.67 (0.43–1.02)
PICC	32	7 (21.9)	0.45 (0.19–1.1)
peripheral cannulae	261	3 (1.1)	2.38 (0.77–7.4)
tunnelled central venous line catheters	10	0 (0)	—
Method of OPAT drug administration		iv catheter-related	iv catheter-related
self-administration	30	8 (27)	12.0 (6.0–23.9)
mixed administration	20	4 (20)	8.5 (3.3–23.5)
nurse-administered	488	27 (5.5)	4.8 (3.3–7.0)

a AE percentages as a proportion of the number of episodes of use for each variable.

b AE rates per 1000 days of OPAT care, unadjusted for other variables.

### Adverse events

Drug-related AEs complicated 13 (2.4%) OPAT episodes at a rate of 1.7 (95% CI 0.9–2.9) per 1000 drug days (Table S5). There was no difference in the AE rate between β-lactam versus non-β-lactam drugs (Table 1). Rash was the commonest drug-related AE, occurring in eight episodes of OPAT care (62% of drug-related AE) (Table S5).

**Figure 1.**
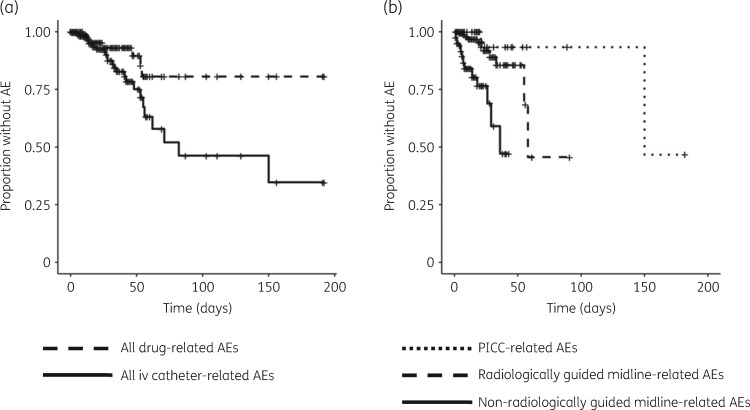
Rate of OPAT-related AEs. Kaplan–Meier plots demonstrating (a) AEs attributed to either drugs or iv catheters and (b) catheter-related AEs stratified by route of iv access. AEs are indicated by censored events on plots.

Catheter-related AEs occurred significantly more frequently than drug-related AEs (*n *=* *39 in total), complicating 32 (5.9%) episodes at a rate of 5.7 (95% CI 4.2–7.9) per 1000 iv catheter days (*χ*^2^ test for difference in the rate of drug- and iv catheter-related AEs: *P *<* *0.001) (Table 1 and Figure 1a). The commonest iv catheter-related AEs were extravasation (13, 33%), blockage (8, 21%) and displacement (8, 21%) (Table S6). In unadjusted analyses, the rate of AEs was highest for non-radiologically guided midline catheters [*n *=* *23, 59% at 15.6 (95% CI 10.3–23.4) per 1000 iv catheter days] (Figure 1b). In unadjusted analyses, self-administration was associated with more frequent iv catheter-related AEs [12.0 (95% CI 6.0–23.9) per 1000 OPAT days] versus nurse-administered [4.8 (95% CI 3.3–7.0)] or mixed administration [8.5 (95% CI 3.3–23.5)] (Tables 1 and S7). In the adjusted Poisson regression analysis both non-radiologically guided midline catheters (HR 8.4, 95% CI 2.4–51.9, *P *<* *0.004) and self-administration (HR 4.15, 95% CI 1.7–9.1, *P *=* *0.007) remained associated with a higher rate of iv catheter-related AEs.

## Discussion

Delivery of any service obligates clinicians to minimize the risks posed to patients. In this study involving more than 500 OPAT episodes, we have shown that, in contrast to previous observations,^10^^,^^11^ iv catheter-related AEs were the main drivers of AEs, whereas drug-related AEs occurred less frequently than previously described.^9^^,^^11^

The higher rate of AEs associated with iv catheters in our cohort was largely driven by the use of midlines in general and specifically midline catheters that are shorter and less secured, but which can be inserted without radiological guidance in the outpatient setting.^14^ Secured iv catheters, such as PICCs and tunnelled central venous catheters require radiological input for insertion, but have lower rates of complications compared with unsecured midlines.^7^^,^^13^^,^^15^^,^^16^ Crucially, previous studies that directly compared drug- and catheter-related AE rates included few patients using midlines for iv access.^10^^,^^11^

We also observed a higher rate of catheter-related AEs for self-administration of OPAT which remained significant after adjusting for the catheter type. Previous studies have not consistently identified an increased risk for self-OPAT,^7^^,^^10^ but our findings overall suggest that the choices of iv access device and method for OPAT drug administration represent a trade-off between elevated AE risks and the convenience associated with non-radiologically guided midline insertion and self-administration.^18^

The drug-related AE rate in our study (1.7 per 1000 OPAT days) was lower than in a recent US-based case series (2.24 per 1000 OPAT days),^9^ in part driven by the use of vancomycin, an antibiotic associated with a high AE rate.^13^ In contrast, teicoplanin, which is not routinely available in the USA, was almost exclusively the glycopeptide of choice in our cohort, supporting its use as an alternative to vancomycin to deliver safe and effective OPAT care.^19^ This is backed up by the high OPAT success rate observed, although it is noteworthy that the majority of OPAT episodes in our cohort commenced after initiation of iv antimicrobial therapy as an inpatient, which may have both impacted significantly on clinical outcomes as well as reduced early drug-related AEs.

Our study had a number of strengths, including a large number of cases and a heterogeneous patient cohort, generalizable to other centres, where the use of iv antimicrobials was subject to physician stewardship both prior to access to OPAT services and during OPAT care. We also observed high OPAT success rates (>85%) comparable with those documented in previous studies.^1^ Nevertheless, this was a cohort study at risk of unrecognized confounding bias despite the prospective and systematic data collection. Secondly, despite the large cohort size, the numbers remained low for each OPAT indication, limiting detailed multivariable analyses, including the interplay between the choice of antimicrobial and the choice of iv catheter. Thirdly, despite the use of national reporting standards for AEs, we cannot exclude gradual reporting bias over time, although this should not have differed between drug- and catheter-related AEs. Finally, catheter-related AE rates were calculated over the entire study period, during which expertise of OPAT staff inserting non-radiologically guided midline catheters may have improved.

In summary, we present evidence from a large prospective cohort of OPAT patients to demonstrate that iv catheter-related AEs exceed those associated with parenteral antimicrobial drugs. As such, we recommend regular and efficient stewardship of iv antimicrobials,^20^ irrespective of class, both at referral to and during OPAT care, in order to minimize the duration of iv catheter use and the consequent harm to patients.

## Supplementary Material

Supplementary Data

## References

[1] Mitchell ED, Czoski Murray C, Meads D et al Clinical and cost-effectiveness, safety and acceptability of *c*ommunity *i*ntra*v*enous *a*ntibiotic *s*ervice models: CIVAS systematic review. BMJ Open 2017; 7: e013560. 10.1136/bmjopen-2016-013560 PMC5775457 28428184

[2] Gilchrist M, Seaton RA. Outpatient parenteral antimicrobial therapy and antimicrobial stewardship: challenges and checklists. J Antimicrob Chemother 2015; 70: 965–70. 25538169 10.1093/jac/dku517

[3] Ponce González MA, Mirón Rubio M, Mujal Martinez A et al Effectiveness and safety of outpatient parenteral antimicrobial therapy in acute exacerbation of chronic obstructive pulmonary disease. Int J Clin Pract 2017; 71: doi:10.1111/ijcp.13022. 10.1111/ijcp.13022 28949430

[4] Suleyman G, Kenney R, Zervos MJ et al Safety and efficacy of outpatient parenteral antibiotic therapy in an academic infectious disease clinic. J Clin Pharm Ther 2017; 42: 39–43. 27747899 10.1111/jcpt.12465

[5] Mujal A, Sola J, Hernandez M et al Safety and effectiveness of outpatient parenteral antimicrobial therapy in older people. J Antimicrob Chemother 2016; 71: 1402–7. 26832749 10.1093/jac/dkv478

[6] Means L, Bleasdale S, Sikka M et al Predictors of hospital readmission in patients receiving outpatient parenteral antimicrobial therapy. Pharmacotherapy 2016; 36: 934–9. 27393717 10.1002/phar.1799

[7] Barr DA, Semple L, Seaton RA. Self-administration of outpatient parenteral antibiotic therapy and risk of catheter-related adverse events: a retrospective cohort study. Eur J Clin Microbiol Infect Dis 2012; 31: 2611–9. 22526869 10.1007/s10096-012-1604-z

[8] Shrestha NK, Mason P, Gordon SM et al Adverse events, healthcare interventions and healthcare utilization during home infusion therapy with daptomycin and vancomycin: a propensity score-matched cohort study. J Antimicrob Chemother 2014; 69: 1407–15. 24398341 10.1093/jac/dkt512

[9] Keller SC, Williams D, Gavgani M et al Rates of and risk factors for adverse drug events in outpatient parenteral antimicrobial therapy. Clin Infect Dis 2018; 66: 11–9. 29020202 10.1093/cid/cix733 PMC5848264

[10] Matthews PC, Conlon CP, Berendt AR et al Outpatient parenteral antimicrobial therapy (OPAT): is it safe for selected patients to self-administer at home? A retrospective analysis of a large cohort over 13 years. J Antimicrob Chemother 2007; 60: 356–62. 17566002 10.1093/jac/dkm210

[11] Gardiol C, Voumard R, Cochet C et al Setting up an outpatient parenteral antimicrobial therapy (OPAT) unit in Switzerland: review of the first 18 months of activity. Eur J Clin Microbiol Infect Dis 2016; 35: 839–45. 26886452 10.1007/s10096-016-2606-z

[12] Panesar P, Jones A, Aldous A et al Attitudes and behaviours to antimicrobial prescribing following introduction of a smartphone app. PLoS One 2016; 11: e0154202. 27111775 10.1371/journal.pone.0154202 PMC4844117

[13] Keller SC, Dzintars K, Gorski LA et al Antimicrobial agents and catheter complications in outpatient parenteral antimicrobial therapy. Pharmacotherapy 2018; 38: 476–81. 29493791 10.1002/phar.2099 PMC5902416

[14] Carroll WD, Anderson M, Reddy RV et al Vascular access in cystic fibrosis—does size matter? J Vasc Access 2005; 6: 72–5. 16552688 10.1177/112972980500600205

[15] Xu T, Kingsley L, DiNucci S et al Safety and utilization of peripherally inserted central catheters versus midline catheters at a large academic medical center. Am J Infect Control 2016; 44: 1458–61. 27908432 10.1016/j.ajic.2016.09.010

[16] Moureau N, Poole S, Murdock MA et al Central venous catheters in home infusion care: outcomes analysis in 50,470 patients. J Vasc Interv Radiol 2002; 13: 1009–16. 12397122 10.1016/s1051-0443(07)61865-x

[17] Marks M, Pollara G, Miller D et al elCID: an electronic Clinical Infection Database to support integrated clinical services and research in infectious diseases. J Infect 2015; 71: 402–5. 25892135 10.1016/j.jinf.2015.04.007 PMC4868136

[18] Vargas-Palacios A, Meads DM, Twiddy M et al Cost-effectiveness of outpatient parenteral antibiotic therapy: a simulation modelling approach. J Antimicrob Chemother 2017; 72: 2392–400. 28505278 10.1093/jac/dkx123 PMC5890745

[19] Nathwani D, Barlow GD, Ajdukiewicz K et al Cost-minimization analysis and audit of antibiotic management of bone and joint infections with ambulatory teicoplanin, in-patient care or outpatient oral linezolid therapy. J Antimicrob Chemother 2003; 51: 391–6. 12562708 10.1093/jac/dkg061

[20] Pollara G, Bali S, Marks M et al Time efficiency assessment of antimicrobial stewardship strategies. Clin Infect Dis 2017; 64: 1463–4. 10.1093/cid/cix220 PMC5411396 28329195

